# The Effectiveness of Warm-Up Using an Assistive Device in Wheelchair Basketball: A Feasibility Study of Able-Bodied Participants

**DOI:** 10.7759/cureus.68751

**Published:** 2024-09-05

**Authors:** Ryo Nakazawa, Kazushi Takahashi, Kazunori Koseki, Kenichi Yoshikawa, Hirotaka Mutsuzaki

**Affiliations:** 1 Department of Physical Therapy, Ibaraki Prefectural University of Health Sciences Hospital, Ami, JPN; 2 Center for Medical Science, Ibaraki Prefectural University of Health Sciences, Ami, JPN; 3 Department of Orthopedic Surgery, Ibaraki Prefectural University of Health Sciences Hospital, Ami, JPN

**Keywords:** able-bodied participants, assistive device, lumbar-type hybrid assistive limb, warm-up, wheelchair basketball

## Abstract

Introduction

A warm-up is often performed to prevent injury and prepare for optimal performance. Nonetheless, research on its impact on performance, particularly in para-sports, remains limited. We hypothesized that the use of an assistive device during warm-up would enable wheelchair basketball players to perform full-body movements efficiently and effectively, contributing to enhanced wheelchair mobility. Therefore, this feasibility study aimed to assess the safety of warm-up exercise with an assistive device and the changes in wheelchair mobility performance before and after warm-up in able-bodied participants.

Methods

Thirteen able-bodied participants (nine males and four females; mean age: 34.3 ± 6.11 years) were analyzed. Warm-up consisted of a five-minute stand-up exercise using the lumbar-type Hybrid Assistive Limb^®^. Before and after warm-up, a 3-3-6 m sprint was performed as a wheelchair mobility performance test. The 3-3-6 m sprint is a test in which the athlete repeatedly accelerates, decelerates, and stops while driving at maximum effort for a total of 12 m (0-3 m, 3-6 m, and 6-12 m). The time required for the 3-3-6 m sprint and maximum instantaneous speed, acceleration time, deceleration time, hip joint angle, and average muscle activity of the lower limb and trunk muscles during the acceleration/deceleration phase of each section were compared before and after warm-up exercise.

Results

Warm-up with an assistive device was safe in healthy participants. The time required for the 3-3-6 m sprint was significantly reduced after the warm-up compared to that before the warm-up (p=0.005). Although not significant, there was a trend toward shorter deceleration times after the warm-up for participants herein.

Conclusions

In able-bodied participants, warm-up with an assistive device is safe; it may improve wheelchair mobility performance. Further research is required to determine its impact on para-athletes with disabilities.

## Introduction

Among parasport events, wheelchair basketball is one of the most popular and has become even more prominent in recent years owing to the Paralympic Games and other international competitions held worldwide [1]. Wheelchair basketball is played by teams composed of people with lower limb amputation or lower-limb/trunk paralysis and requires athletic skills (e.g., passing and shooting on a wheelchair), wheelchair-driving skills (e.g., rapid acceleration, deceleration, stopping, and turning), and repetition of intermittent rapid acceleration and deceleration, especially during games [2,3]. In wheelchair basketball, players are classified from 1 to 4.5 based on their physical condition. A player with a score of 1 has no control of his/her trunk, while a player with a score of 4.5 has trunk function similar to that of an able-bodied player [4]. Recently, the level of competition required has increased as the interest in the sport has grown.

Warm-up is often performed to prevent injury and prepare for optimal performance [5,6]. The general purpose of a warm-up before exercise is to increase the flexibility of muscles and tendons, stimulate the blood flow to the periphery, increase the muscle temperature, and enhance free and harmonious movement [7]. The mechanisms of warm-up include passively increasing and maintaining the muscle temperature and heat-independent psychological effects [6]. Active warm-up, including short aerobic sessions and four to five activations, followed by post-activation strength training and small-sided games, has been shown to enhance performance [8]. While warm-up is considered necessary for optimal performance, there is scant scientific evidence to support its effectiveness. A systematic review and meta-analysis [9] of evidence pertaining to performance improvement using warm-up showed that warm-up improved the performance in 79% of the standards examined and that performance improvement could be demonstrated after the completion of appropriate warm-up activities. Nevertheless, only a few randomized controlled trials are available. Therefore, more studies are required to further determine the role of warm-up in performance improvement. Furthermore, while some studies have investigated the effects of warm-up on the performance of able-bodied athletes in different competitive events [8], only a few studies have reported the effects of warm-up on the performance of wheelchair athletes [10]. Importantly, wheelchair basketball athletes with disabilities include those who have difficulty in performing whole-body exercises and are restricted to exercises that they can perform during warm-up.

We hypothesized that the use of an assistive device (AD) by wheelchair basketball athletes during warm-up would enable them to perform whole-body exercises efficiently and effectively, ultimately contributing to improved wheelchair mobility. Previous studies targeting locomotor disorders have shown that chair stand-up exercises are useful for improving walking speed, dynamic body balance, and muscle strength compared to baseline values [11,12]. Thus, stand-up exercise can be expected to be effective in activating physical activities and improving muscle strength and balance ability, and can be practiced without taking up much space as long as a chair or stand to sit on is available. Additionally, they do not involve transportation, which can be difficult for people with disabilities. Therefore, we focused on stand-up exercises for athletes who require effort to stand up.

The present feasibility study aimed to observe changes in wheelchair mobility performance after warm-up with an AD in able-bodied individuals prior to athletes with impaired lower-limb and trunk function.

## Materials and methods

Participants

This study included 13 healthy adults (nine males and four females). For male participants, the mean age, height, and weight were 34.44 ± 6.86 years, 172.89 ± 2.64 cm, and 69.4 ± 7.46 kg, respectively. For female participants, the mean age, height, and weight were 34.0 ± 1.67 years, 156.75 ± 3.77 cm, and 53.75 ± 3.92 kg, respectively. Table 1 summarizes the participant characteristics. Three participants had at least one year of experience in playing wheelchair basketball.

**Table 1:** Participant characteristics M - male, F - female; a - experience in wheelchair basketball (1+ years); b - history of fractures, other injuries, or surgeries

Participant	Sex	Age (years)	Height (cm)	Weight (kg)	Experience^ a^	Injuries or surgeries ^b^
1	F	35	160	60	No	No
2	F	31	161	55	Yes	Yes
3	M	36	176	75	No	Yes
4	M	38	171	80	No	No
5	M	42	176	79	No	Yes
6	M	30	173	60	No	No
7	M	34	175	58	No	Yes
8	F	34	153	48	No	No
9	M	23	173	73	Yes	Yes
10	M	23	167	60	No	Yes
11	F	36	153	52	No	Yes
12	M	41	173	69	No	No
13	M	43	172	70	Yes	No
Mean ± SD	M	34.44 ± 6.86	172.89 ± 2.64	69. 40 ± 7.46		
	F	34.0 ± 1.67	156.75 ± 3.77	53.75 ± 3.92		
	All	34.3 ± 6.11	167.92 ± 8.04	64.5 ± 10.02		

The inclusion criteria comprised adult healthy participants aged 18 years or older, regardless of sex or history of wheelchair sports competition. The exclusion criteria included individuals with serious complications, such as cardiac, liver, or renal conditions, that hindered exercise participation, as well as those with limited comprehension. A poster explaining the recruitment requirements for the study was used to recruit participants.

Research procedure

**Figure 1:**
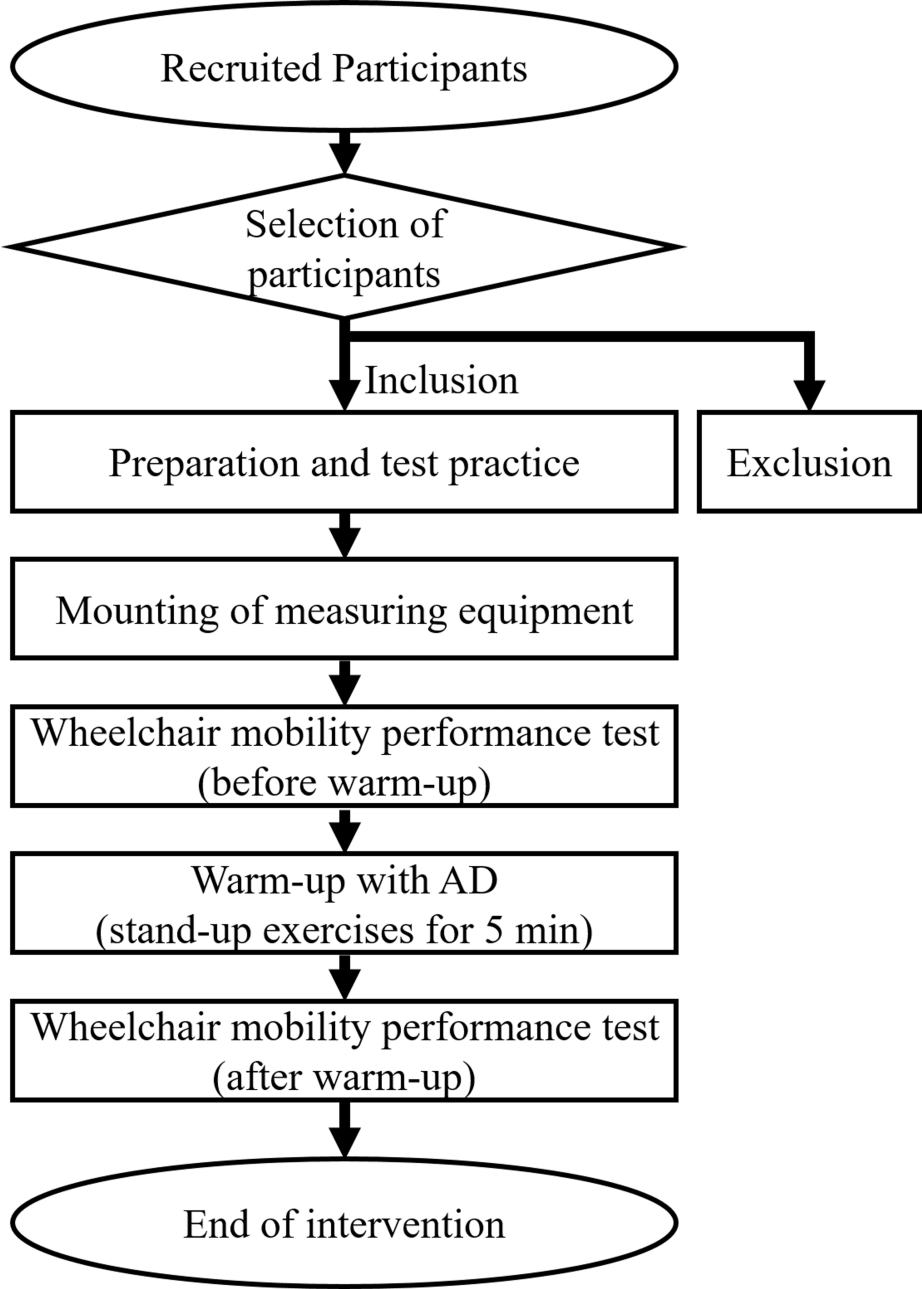
Research procedure AD - assistive device

The following protocol was used to determine the safety of warm-up exercises with an AD and the effect of warm-up on wheelchair mobility performance. All participants were measured according to the schedule in Figure 1. In this study, the lumbar-type Hybrid Assistive Limb® (HAL) was used as the AD for the warm-up. First, all participants underwent a preparatory exercise in advance to understand the performance test. A performance test was then performed, followed by the warm-up described below using lumbar-type HAL. Finally, the performance test was repeated. As a general principle, the performance test was performed once; however, if there was any kind of measurement deficiency, such as noise detection or electrode detachment, the test was repeated two or three times until the measurements had been concluded successfully.

Lumbar-type HAL

**Figure 2:**
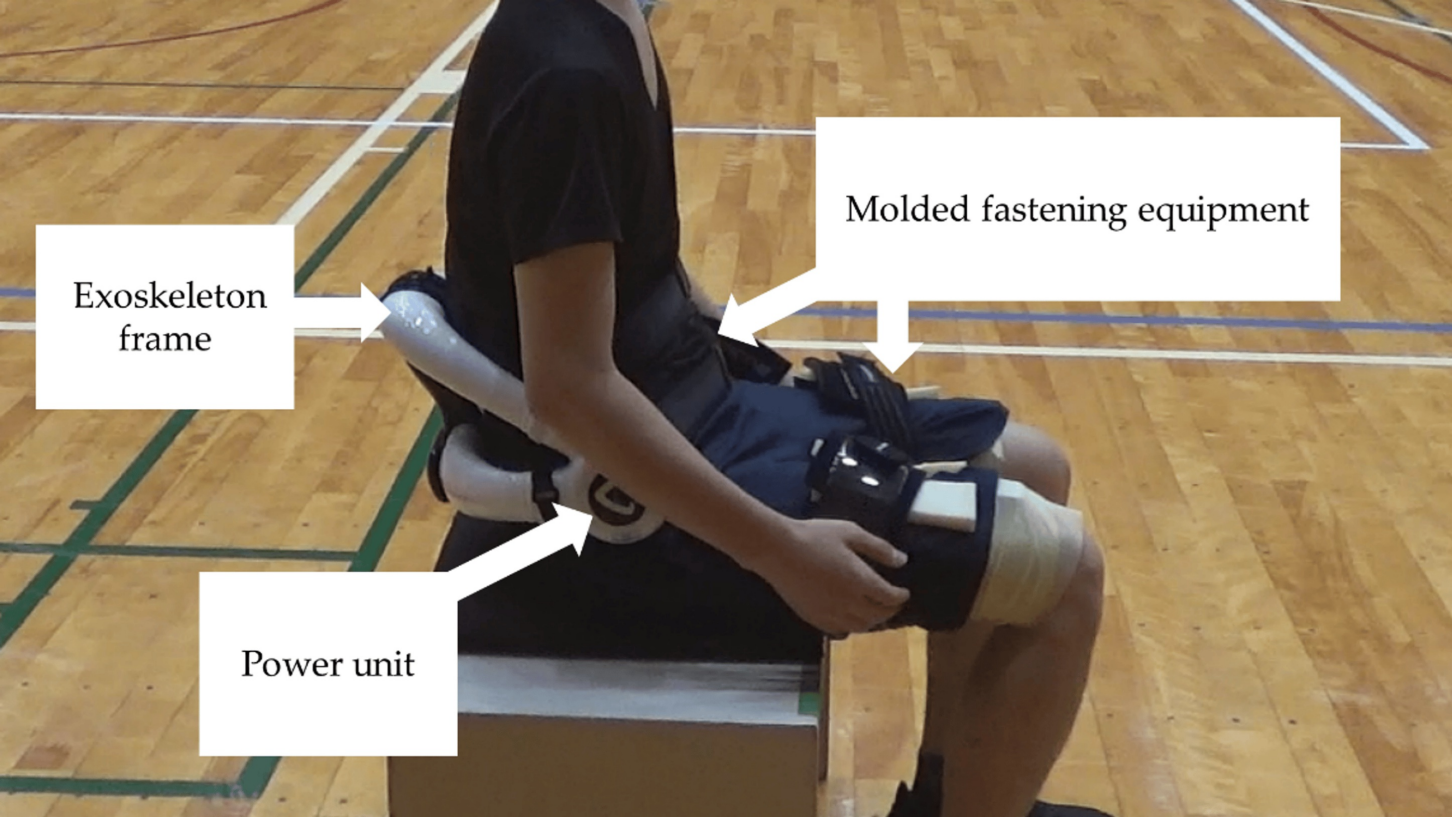
Details of the lumbar-type HAL The sensors were affixed to the lumbar erector spinae. The person in the figure is a research member, not a participant. HAL - Hybrid Assistive Limb®

The HAL is a wearable cyborg (exoskeleton robot) that can detect bio-electrical signals from electrodes attached to the wearer's skin and can assist in movement according to the wearer's will [13]. The lumbar-type HAL consists of a power unit, exoskeleton frame, sensors, and controller (Figure 2). The exoskeleton frame is fixed to the wearer's body by molded fastening equipment at the waist and thighs [14]. The lumbar-type HAL has two control systems: Cybernics Voluntary Control (CVC) and Cybernics Autonomous Control (CAC) [14]. The CVC system detects the trunk extension motion of the wearer through bioelectrical signals and assists the joint torque in response to voluntary muscle activity, whereas the CAC system uses sensors to support moments in the lumbar region caused by trunk flexion. The torque tuner allows the lumbar type to adjust the amount of assistance to five levels.

Among several types of HALs, the medical lower-limb type is used in exercise therapy for neuromuscular diseases [15] and spinal cord injuries [16] or after total knee arthroplasty [17], whereas the single-joint type is used for spinal cord injuries [18] and in children with cerebral palsy [19]. By attaching sensors to the lumbar erector spinae muscles, the lumbar-type HAL can assist in lumbar spine flexion, extension, and standing movements according to the wearer's will. Previous studies using the lumbar-type HAL in able-bodied participants have reported reduced worry load during squatting exercises [20].

Warm-up protocol with the lumbar-type HAL

**Figure 3:**
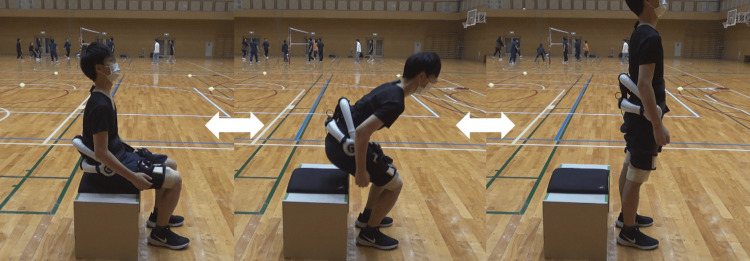
Stand-up exercise with the lumbar-type HAL Participants performed five minutes of stand-up exercise at an arbitrary speed. The person in the figure is a research member, not a participant. HAL - Hybrid Assistive Limb®

The participants wore the HAL in an edge-sitting position with their hip and knee joints at approximately 90°. Referring to previous research, participants performed a repeated stand-up exercise for five minutes [20,21] (Figure 3). At this time, the participants were allowed to take a break, depending on their fatigue, and were instructed to repeat the exercise at any rhythm and speed. Based on previous studies, the HAL was set up in hybrid (CVC) mode [15,17]. Five assist levels were optimally set to enable each participant to sit comfortably. The participants practiced beforehand to determine the amount of assistance. To ensure exercise safety and check for the presence of adverse events, the pulse rate was measured before and after the exercise, the fatigue level (Borg scale) was assessed, and participant movements were observed.

Outcome Measures

Each of the parameters described below was measured before and after the warm-up with an AD, and changes were compared. The measurement items were as follows: wheelchair mobility performance test (The 3-3-6 m sprint), hip joint angle, and electromyography patterns.

Wheelchair mobility performance test

**Figure 4:**
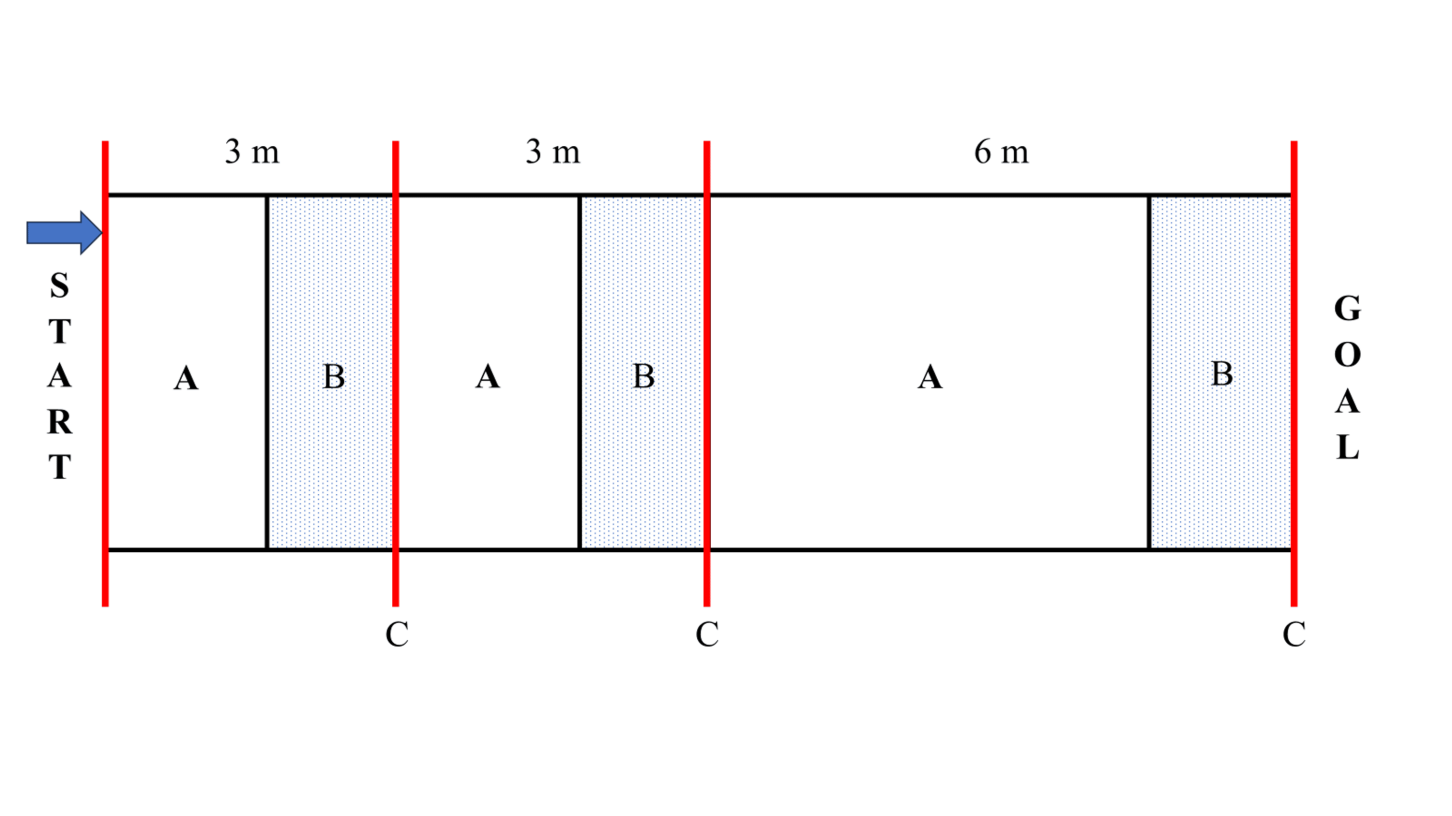
Details of the 3-3-6 m sprint A - acceleration time (acceleration phase); B - deceleration time (deceleration phase); C - stop point

The 3-3-6 m sprint was employed. The 3-3-6 m sprint is a test of maximum drive with repeated acceleration and deceleration at 3 m, 3 m, and 6 m sections; at the 3 m, 6 m, and 12 m points, the wheelchair must come to a complete stop at each stop point (Figure 4). This test is part of the assessment of wheelchair mobility performance in wheelchair basketball, and its validity and reliability have been confirmed [22]. In each section (0-3 m, 3-6 m, and 6-12 m), the acceleration time (acceleration phase: AP) was defined as the time from the point where the handrim was grasped for driving until the maximum instantaneous speed was reached. The deceleration time (deceleration phase: DP) was defined as the time until the wheelchair came to a complete stop after achieving the maximum instantaneous speed. The participants performed preparatory exercises and test practice before the test was started. The time required for the 3-3-6 m sprint was measured using a stopwatch. A video (60 fps) from the sagittal plane was captured to verify the drive start and stop points. Experienced participants who owned their own wheelchairs were allowed to use them. The remaining participants used the best-fitting wheelchair we had, which was designed for basketball use. Each participant consistently used the same wheelchair before and after the measurement.

Three-Dimensional Motion Analysis and Electromyography (EMG)

**Figure 5:**
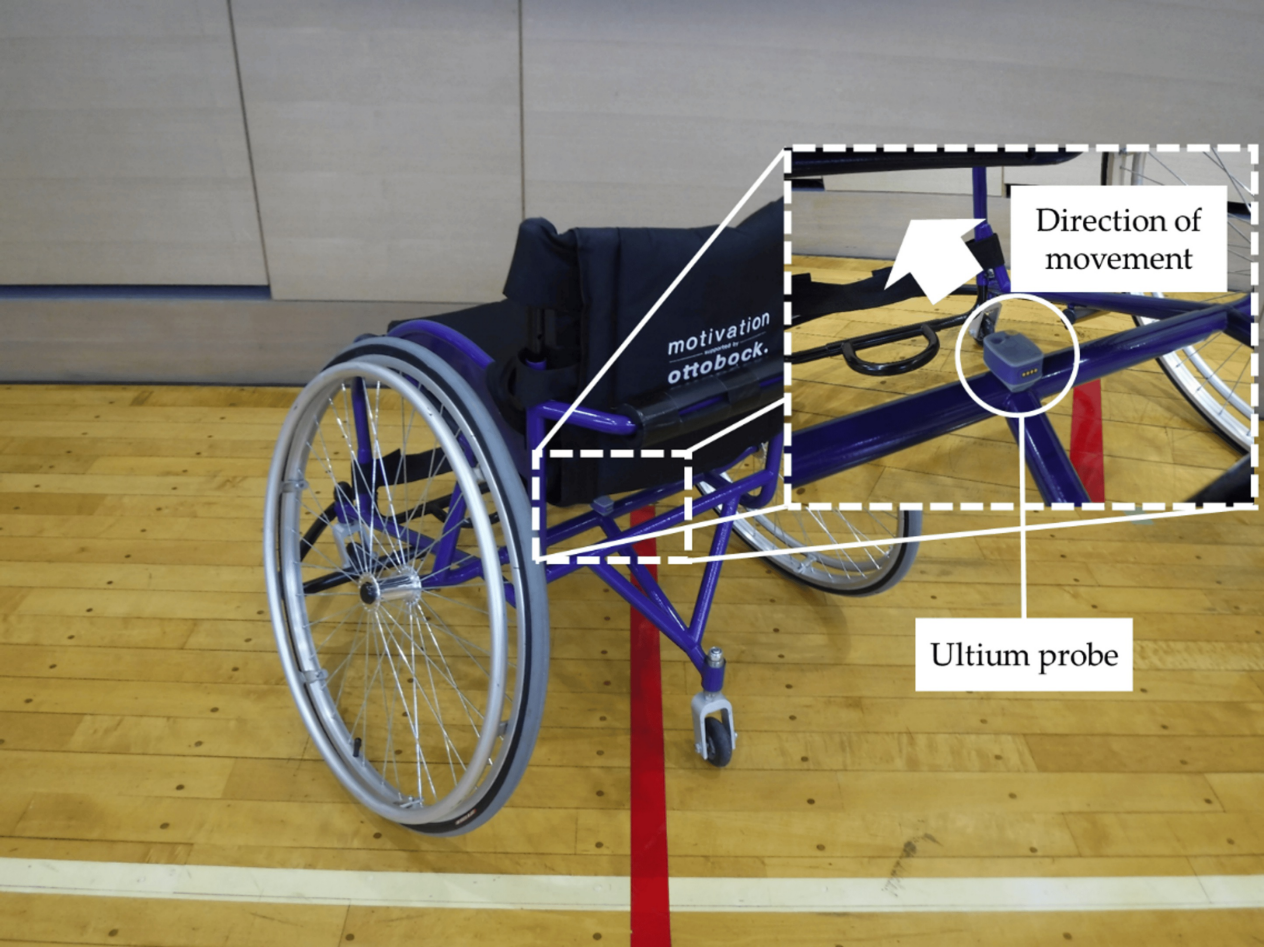
Acceleration measurement in practice The Ultium probe was firmly taped in place

We used myoMOTION (Noraxon USA, Scottsdale, AZ, USA), a wireless inertial measurement unit (IMU) system consisting of a receiver and five IMUs, to perform three-dimensional motion analysis during wheelchair mobility and to evaluate the hip joint angle of the participants during wheelchair mobility. Each IMU was positioned on a body segment according to the C7, L1, sacral, and both femoral regions provided by the IMU system software (myoRESEARCH version 3.16.86; Noraxon USA, Scottsdale, AZ, USA). Each IMU had a local coordinate system and measured acceleration. Prior to measurements, the system was calibrated with the participants in a seated position. The change in hip joint angle was visually confirmed using the peak value of the waveform, braking start point, maximum extension position during the DP, and overall shape. Wheelchair acceleration was measured using the Ultium EMG sensor system (Noraxon USA, Scottsdale, AZ, USA). The Ultium probe had a built-in acceleration sensor attached to the axle of the wheelchair to measure acceleration in the direction at which the wheelchair was moving (Figure 5). The maximum instantaneous velocity of each section was calculated from the acceleration data using myoRESEARCH software. Subsequently, the time was recorded. Additionally, video images synchronized with the IMU system were used to visually determine the drive start and stop points for each section and to measure the acceleration and deceleration times. EMG patterns of the bilateral rectus abdominis, rectus femoris, latissimus dorsi, and lumbar erector spinae muscles were recorded, and the raw waveform of EMG was depicted. The electrode attachment position for each muscle was based on the electrode placement site recommended by surface EMG for non-invasive assessment of muscle [23]. Before placing the EMG surface electrodes on the participants, the skin surface was shaved, polished, and cleaned with an alcohol swab to improve the contact between the skin and electrodes and reduce skin impedance. EMG was set at a sampling frequency of 2000 Hz and bandpass filter of 10-500 Hz and was normalized to the maximum value measured before warm-up at 100%. The EMG data were then rectified and smoothed (root mean square algorithm, smoothing window width: 100 ms).

Statistical analysis

Statistical analysis for changes in performance before and after warm-up was performed using SPSS version 25 (IBM Inc., Armonk, NY, USA). The time required for the 3-3-6 m sprint, maximum instantaneous speed for each section, acceleration time for each section, deceleration time for each section, and mean EMG difference for each muscle in each section were compared before and after warm-up. The normality of each measured parameter was checked, and the paired t-test was used to evaluate normality and detect differences in means. For non-normally distributed parameters, the Wilcoxon signed-rank test was used to compare median differences. Statistical significance was set at the 5% level for both means and medians. Each variable was checked for normality, and the mean was compared for variables with normal distributions and the median for variables with non-normal distributions. The standard deviation (SD) was examined for differences in means, while the interquartile range (IQR) was examined for differences in medians. In addition, effect sizes (r) were calculated, with 0.1 indicating a small effect, 0.3 indicating a medium effect, and 0.5 or greater indicating a large effect [24].

## Results

The changes in measured wheelchair mobility performance before and after warm-up are shown in Table 3. The mean time required for the 3-3-6 m sprint was reduced from 9.30 ± 1.52 s to 8.84 ± 1.40 s (p=0.005, r=0.70). The mean deceleration time changed from 0.70 ± 0.24 s to 0.62 ± 0.17 s in the 0-3 m section (p=0.06, r=0.52) and from 1.09 ± 0.32 s to 0.94 ± 0.20 s in the 6-12 m section (p=0.11, r=0.45).

**Table 2:** Assessment results before and after warm-up Values are shown as mean ± standard deviation or median (interquartile range) **: Significant difference (p<0.01), †: Small effect, ††: Medium effect, †††: Large effect

Section	Outcome measures		Before-warm up	After-warm up	p-value	Effect size (r)
All	12 m duration	(sec)	9.30 ± 1.52	8.84 ± 1.40	0.005^**^	0.7 ^†††^
0-3 m	Maximum instantaneous speed	(m/s)	2.22 ± 0.56	2.27 ± 0.42	0.57	0.17 ^†^
	Acceleration time	(sec)	1.80 ± 0.23	1.83 ± 0.22	0.64	0.14 ^†^
	Deceleration time	(sec)	0.70 ± 0.24	0.62 ± 0.17	0.06	0.52 ^†††^
3-6 m	Maximum instantaneous speed	(m/s)	2.28 ± 0.61	2.29 ± 0.52	0.92	0.03
	Acceleration time	(sec)	1.56 (1.37-1.77)	1.51 (1.43‐1.57)	0.46	0.21 ^†^
	Deceleration time	(sec)	0.70 ± 0.16	0.68 ± 0.23	0.67	0.12 †
6-12 m	Maximum instantaneous speed	(m/s)	2.73 ± 0.97	2.74 ± 0.69	0.93	0.02
	Acceleration time	(sec)	2.71 ± 0.49	2.71 ± 0.43	0.91	0.03
	Deceleration time	(sec)	1.09 ± 0.32	0.94 ± 0.20	0.11	0.45 ^††^

As the data from the participants' left side contained artifacts and electrode detachment, each EMG was observed on the right side. Similarly, the right side value was used for the hip joint angle. One cycle (0-100%) was defined as acceleration, deceleration, and stopping from the drive start point for each of the three sections.

**Figure 6:**
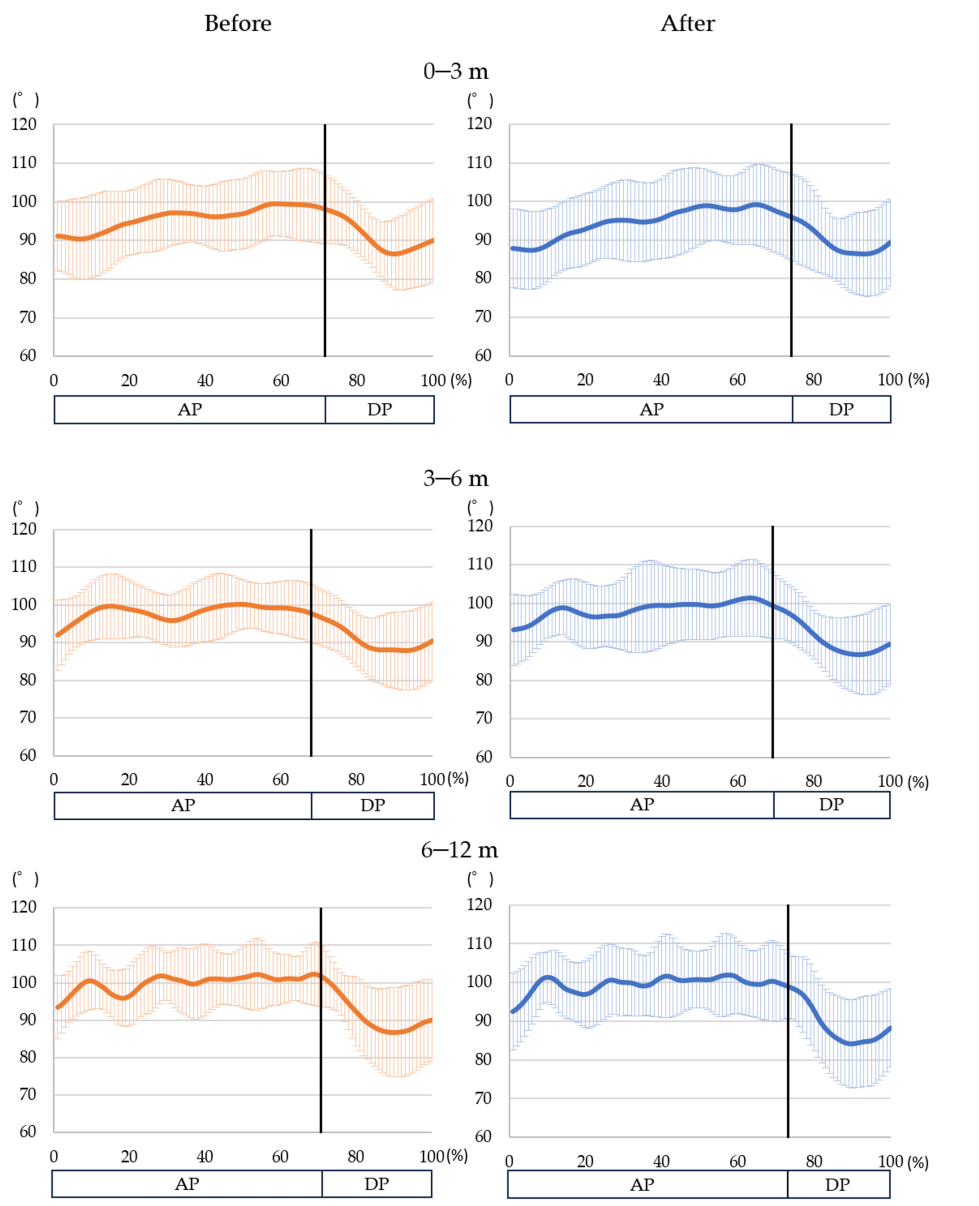
Change in the hip flexion angle Only the values for the right hip joint are noted. The Y-axis represents the mean right hip flexion angle. The X-axis shows the percentage of time in each section. The solid line in each waveform shows the change in the right hip flexion angle, and the shaded area represents the standard deviation. Before - before warm-up; After - after warm-up; AP - acceleration phase; DP - deceleration phase

The right hip angle did not change significantly enough to exceed the SD in the performance tests before and after warm-up (Figure 6). The mean (± SD) or median (IQR) EMG activity for each section before and after the warm-up are shown in Table 4. The following changes in EMG showed more than medium effect sizes. In the 0-3 m section, the rectus abdominis increased from 21.77 ± 8.64 to 23.52 ± 8.36 (p=0.26, r=0.32) and the latissimus dorsi increased significantly from 10.60 (9.26-19.00) to 13.40 (10.40-19.60; p=0.02, r=0.55) in the AP. The rectus abdominis decreased from 8.73 ± 4.35 to 7.71 ± 2.96 (p=0.27, r=0.32) in the DP. In the 3-6 m section, the latissimus dorsi increased from 14.70 (9.97-18.70) to 12.70 (10.90-21.60; p=0.20, r=0.35) and the lumbar erector spinae increased from 9.79 ± 4.98 to 10.4 ± 5.15 (p=0.15, r=0.41) in the AP. The rectus abdominis decreased from 7.19 (5.75-9.98) to 5.81 (5.18-9.44; p=0.09, r=0.44) and the rectus femoris decreased from 20.43 ± 15.58 to 17.50 ± 12.42 (p=0.17, r=0.39) in the DP. In the 6-12 m section, the rectus abdominis decreased significantly from 7.61 (5.78-11.00) to 5.83 (5.04-6.72; p=0.04, r=0.51) in the DP.

**Table 3:** Results of EMG activity before and after warm-up All muscle activity values are for the right side only. Values are shown as the mean ± standard deviation or median (interquartile range) AP - acceleration phase; DP - deceleration phase; EMG - electromyography *: Significant difference (p<0.05), †: Small effect, ††: Medium effect, †††: Large effect

Section	EMG (%)		Before-warm up	After-warm up	p-value	Effect size (r)
0-3 m	Rectus abdominis	AP	21.77 ± 8.64	23.52 ± 8.36	0.26	0.32 ^††^
		DP	8.73 ± 4.35	7.71 ± 2.96	0.27	0.32 ^††^
	Rectus femoris	AP	11.49 ± 5.84	11.43 ± 5.16	0.96	0.01
		DP	15.78 ± 11.31	15.52 ± 9.24	0.85	0.06
	Latissimus dorsi	AP	10.60 (9.26–19.00)	13.40 (10.40–19.60)	0.02*	0.55 ^†††^
		DP	23.76 ± 13.93	26.24 ± 14.48	0.39	0.25 ^†^
	Lumbar erector spinae muscles	AP	8.5 ± 5.68	8.51 ± 4.48	0.99	0.00
		DP	17.43 ± 12.96	16.19 ± 11.20	0.39	0.25 ^†^
3-6 m	Rectus abdominis	AP	22.27 ± 7.41	21.95 ± 7.55	0.84	0.06
		DP	7.19 (5.75–9.98)	5.81 (5.18–9.44)	0.09	0.44 ^††^
	Rectus femoris	AP	19.12 ± 10.83	20.06 ± 10.14	0.57	0.17 ^†^
		DP	20.43 ± 15.58	17.50 ± 12.42	0.17	0.39 ^††^
	Latissimus dorsi	AP	14.70 (9.97–18.70)	12.70 (10.90–21.60)	0.20	0.35 ^††^
		DP	24.39 ± 11.00	25.74 ± 15.09	0.58	0.16 ^†^
	Lumbar erector spinae muscles	AP	9.79 ± 4.98	10.4 ± 5.15	0.15	0.41 ^††^
		DP	16.06 ± 10.58	16.94 ± 11.56	0.41	0.24 ^†^
6-12 m	Rectus abdominis	AP	23.1 ± 7.31	24.52 ± 8.33	0.45	0.22 ^†^
		DP	7.61 (5.78–11.00)	5.83 (5.04–6.72)	0.04*	0.51 ^†††^
	Rectus femoris	AP	24.53 ± 13.97	24.67 ± 12.51	0.96	0.02
		DP	21.12 ± 16.09	17.57 ± 13.10	0.34	0.28 ^†^
	Latissimus dorsi	AP	17.93 ± 10.41	18.3 ± 11.10	0.44	0.23 ^†^
		DP	23.97 ± 14.44	22.78 ± 14.12	0.59	0.16 ^†^
	Lumbar erector spinae muscles	AP	10.36 ± 4.52	10.70 ± 5.73	0.61	0.15 ^†^
		DP	17.29 ± 11.87	17.28 ± 12.41	1.00	0.00

## Discussion

In the present study, warm-up with an AD was safely performed in healthy participants, and changes in wheelchair mobility performance were observed. Wheelchair mobility performance improved after five minutes of stand-up exercise using an AD for able-bodied participants, suggesting that the use of an AD allowed the warm-up to continue without excessive fatigue, possibly contributing to the subsequent improvement in performance. In fact, the mean number of times participants stood up was 96.56 ± 24.14, reaching approximately 100 times, and after warm-up, the participants' mean Borg scale value increased from 8.78 ± 1.69 to 12.7 ± 1.63, with a mean increase in HR of 38%. This warm-up exercise was generally performed at the anaerobic threshold level owing to the use of the AD. The exercise was performed at an arbitrary speed rather than at maximum effort without significant energy depletion, which could be particularly effective during the introductory portion of the warm-up period. As shown by prior research, performing a warm-up at approximately 40-60% VO2max for 5-10 min, followed by five minutes of recovery, improves short-term performance [25]. We anticipate that para-athletes who have difficulty standing up will find it easier to perform the exercise with the AD, which will reduce over-exertion. Increases in the hip extension angle and earlier maximum extension position during the DP were expected. This is because the lumbar-type HAL assists with the wearer's trunk and hip extension. However, the expected changes were not achieved. The magnitude of trunk and hip motion did not appear to be affected. It is possible that the participants in this study were able-bodied participants who were adapted to the exercise to some degree during the preparation and practice phase and were able to generate sufficient hip motion at the time of the before warm-up performance test. Therefore, it is possible that no change was observed before and after warm-up. It was also expected that acceleration/deceleration times would decrease and muscle activity would increase, but this was not clear due to the variability among participants in this study.

In this study, the lumbar-type HAL was used as the AD. Previous studies using the lumbar-type HAL in able-bodied participants have reported reduced worry load during squatting exercises [19]. In this study, we believe that the participants' movements were safely facilitated by adjusting the amount of assistance. The lumbar-type HAL used in this study weighs only approximately 3 kg, including batteries, and is lighter and more portable than the lower-limb type HAL, potentially allowing the wearer to wear it alone. A previous study found that the wearing time for the lumbar-type HAL was approximately five minutes when performed by the wearer themselves [26]. In this study, it was possible to fit it within five minutes at least. Consequently, it is relatively easy to wear and can be easily incorporated into pre-competition warm-up. It can also be employed for re-warm-up before a wheelchair basketball game or during the short half-time period in a game.

A previous study suggested that trunk muscle activity is essential for wheelchair mobility and reported an increase in this activity during acceleration [27]. The present study also showed an increase in mean muscle activity in the rectus abdominis muscle at 0-3 m in the AP. Although this change was not statistically significant, a medium effect size was observed. Similarly, a moderate increase in lumbar erector spinae muscle activity was observed at 3-6 m in the AP, also with a moderate effect size. However, there were sections where the activity of these two muscles did not increase, precluding a definitive conclusion. No significant change or effect on maximum instantaneous speed or acceleration time was observed in each section, suggesting that the effect on wheelchair mobility acceleration was small in this study.

EMG in the DP showed a significant decrease in rectus abdominis muscle activity in the 6-12 m section, as well as medium effect sizes, although not significant, in the other two sections (0-3 m, 3-6 m), suggesting that reduced rectus abdominis muscle activity is responsible for the reduced deceleration time in the participants in this study. Participants in the DP extend their trunks to brake their wheelchairs, and decreased rectus abdominis muscle activity, the antagonist muscle, likely facilitates this movement. Although there was no significant change in the activity of the erector spinae muscles in the lumbar region in all three sections, the decrease in rectus abdominis activity during trunk extension may have resulted in more efficient exercise, even though the muscle activity was the same as before the warm-up. The stand-up exercise with the lumbar-type HAL is similar to the trunk and hip movements during wheelchair-mobility acceleration and deceleration movements. It is possible that the repetition of the stand-up exercise might have optimized the muscle activity of the trunk and lower limbs, resulting in decreased muscle activity even when the same task was performed. It has been reported that the HAL can assist with the wearer's voluntary movements in real-time and that this feedback can be used to adjust antagonistic and cooperative joint movements [28]. Although there were no significant differences in the maximum instantaneous speed or acceleration/deceleration time in each section, the improved performance in wheelchair mobility can be attributed to the fact that the trunk and lower limb exercises using the lumbar-type HAL facilitated optimal muscle activity for the task and dynamic hip and trunk movement, especially during the DP. These results suggest that performing stand-up exercises using an AD may be an effective warm-up for wheelchair basketball, especially for improving agility during wheelchair mobility.

This study has several limitations. First, the results varied between participants due to previous wheelchair sports experience, wheelchair fitting, and learning effects. In particular, participants who did not have previous wheelchair sports experience may have been particularly affected by learning effects. Additionally, the wheelchairs used by several participants in this study for basketball were not perfectly fitted to their individual physiques. Future studies with larger sample sizes are needed to address the variability in results. Second, this study did not disaggregate the data by sex. Therefore, there may be sex-based differences in results. Third, the present study found no clear trend in joint angle or EMG changes. Hence, it was not possible to define the factors contributing to the change in performance. Other joint angles and muscle activities should be measured to examine their influence on performance changes. Further, more detailed studies with a larger number of participants are required in the future to ascertain the factors that influence the performance of wheelchair mobility.

## Conclusions

In this study, a five-minute stand-up exercise using an AD was used as a warm-up. The warm-up was safely performed among healthy participants, and the time required for the 3-3-6 m sprint after warm-up was significantly reduced, suggesting potential enhancement in wheelchair mobility following AD warm-up. However, further research is needed, as several participants had limited experience with wheelchair sports, and performance alterations may be influenced by a learning effect.
